# Nucleic acid direct delivery to fibroblasts: a review of nucleofection and applications

**DOI:** 10.1186/s13036-022-00309-5

**Published:** 2022-11-04

**Authors:** Ranyue Ren, Jiachao Guo, Guangwu Liu, Hao Kang, Hans-Günther Machens, Arndt F. Schilling, Alex Slobodianski, Ziyang Zhang

**Affiliations:** 1 Department of Orthopedics, Tongji Hospital, Tongji Medical College, Huazhong University of Science and Technology, Wuhan, Hubei China; 2 Department of Pediatric Surgery, Tongji Hospital, Tongji Medical College, Huazhong University of Science and Technology, Wuhan, Hubei China; 3 Department of Plastic Surgery and Hand Surgery, Faculty of Medicine, Klinikum Rechts Der Isar, Technische Universität München, Munich, Germany; 4 Department of Trauma Surgery, Orthopaedics and Plastic Surgery, University Medical Center Göttingen, Göttingen, Germany

**Keywords:** Nucleofection, Fibroblasts, Gene therapy, Transfection efficiency, Clinical applications

## Abstract

The fibroblast is one of the ideal target cell candidates for cell-based gene therapy approaches to promote tissue repair. Gene delivery to fibroblasts by viral transfection has been confirmed to have high transfection efficiency. However, in addition to immunogenic effects of viruses, the random integration of viral genes may damage the genome, affect the cell phenotype or even cause cancerous mutations in the transfected cells. Due to these potential biohazards and unknown long-term risks, the clinical use of viral transfection has been very limited. In contrast, initial non-viral transfection methods have been simple and safe to implement, with low immunogenicity, insertional mutagenesis, and risk of carcinogenesis, but their transfection efficiency has been relatively low. Nucleofection, a more recent non-viral transfection method, now combines the advantages of high transfection efficiency and direct nucleic acid delivery to the nucleus with a high safety.

Here, we reviewed recent articles on fibroblast nucleofection, summarized different research points, improved methods and application scopes, and opened up ideas for promoting the further improvement and development of fibroblast nucleofection to meet the needs of a variety of disease research and clinical applications.

## Background

Fibroblasts are the most common cells in connective tissue, and are differentiated from mesenchymal cells. The morphology and function of fibroblasts often undergo dynamic changes during the processes of disease development and tissue repair [1]. Fibroblasts can easily respond to the signals sent out from the surrounding tissue, and have excellent plasticity. They can aggregate and accumulate in injured tissue and surgical areas [2–4], and are mainly involved in the proliferation and remodeling phases of damaged tissue repair [5]. Fibroblasts are rich in rough endoplasmic reticulum, free ribosomes and developed Golgi apparatus, and thus have splendid functions of synthesizing and secreting proteins. They can also deposit extracellular matrix (ECM) and collagen fibers in connective tissue, form granulation tissue together with newborn capillaries to fill tissue defect and provide the basic conditions for the coverage by epidermal cells [1, 6–9]. Fibroblasts are also capable of secreting collagenase for remodeling of repaired tissue. Wounds are always accompanied by varying degrees of cellular degeneration, necrosis, and tissue defects. Subsequent tissue repair and reconstruction is a complex multi-step process that can be summarized into three highly interrelated and overlapping stages: inflammation, proliferation and remodeling [10]. In the inflammatory stage, inflammatory cells are recruited to the damaged parts to resist infection, and secrete chemokines to recruit cells involved in the next stage; in the proliferation stage, various cells migrate, proliferate, and secrete extracellular matrix to participate in tissue regeneration; finally, the extracellular matrix is remodeled and the remodeling stage is completed [5]. When these three stages cannot be fulfilled normally, the wound healing process may be prolonged, or a non-healing status may evolve. For instance, in the presence of certain chronic diseases, the local low expression and rapid degradation of pro-healing proteins and growth factors lead to non-healing wounds, limb ischemia leads to ulcers, gangrene and even amputation, hereditary skin fragility disease results in skin destruction, erosion and infection [11, 12], protracted wounds bring serious physical, mental and financial burdens to patients. Damaged tissue is easily accessible, so gene therapy is well suited for promoting tissue repair and reconstruction [5]. Furthermore, fibroblasts are easily isolated, robust, and grow rapidly in vitro, the properties make them ideal target cell candidates for cell-based gene therapy approaches [13].

**Fig. 1:**
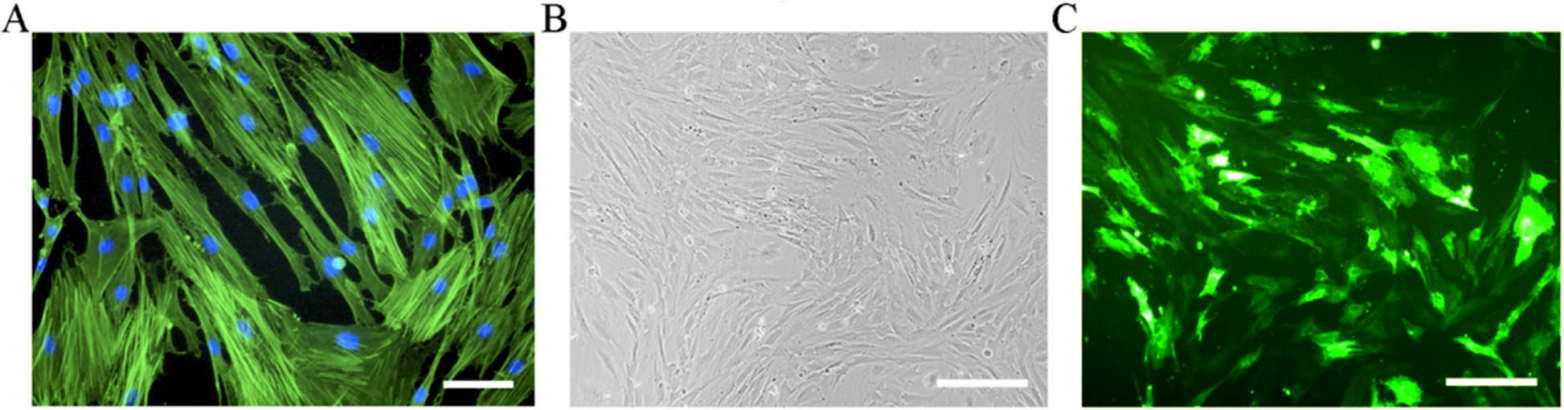
**A** Morphology of rat dermal fibroblasts was analyzed by phalloidin (green) and DAPI (blue) staining. Scale bar represents 100 µm. **B** Rat dermal fibroblasts 6 days after nucleofection, light microscopy. Scale bar represents 200 μm. **C** Rat dermal fibroblasts 6 days after nucleofection, fluorescent microscopy. Scale bar represents 200 μm (author’s unpublished data)

Gene therapy utilizing fibroblasts has been proven feasible by in vitro and in vivo experiments. For example, primary fibroblasts modified by gene nucleofection were used to treat a rat hindlimb ischemia model. They promoted the formation of collateral vessels in the lower limbs of rats and the reconstruction of circulation in the ischemic area [11]. Lwin et al. conducted a phase I clinical trial using gene-modified autologous fibroblast cell therapy in patients with recessive dystrophic epidermolysis bullosa (RDEB) and confirmed the potential curative effect of this therapy on RDEB [12]. Teklemariam et al. nucleotransfected fibroblasts with genes related to immune tolerance and found that they acquired reduced alloreactivity, confirming the clinical application potential of this approach in allogeneic transplantation [14]. A variety of transfection methods have been developed for gene delivery to fibroblasts. Transfection by using viral vectors has been confirmed by a large amount of studies to be feasible and have high transfection efficiency [11, 15, 16]. It has also been reported that after the viral gene is integrated, the encoded gene will be long-term expressed [13]. However, the random integration character of viral genes may disrupt the genome, alter endogenous gene expression and thereby affect cell phenotype or even cause cancerous mutations in transfected cells [17, 18]. Potential biohazards due to immunogenicity of the viral vectors and unknown long-term risks, prohibited transfection therapy using virus as a vector [11, 15]. In comparison, non-viral transfection methods are simple and safe to implement. They can function in the form of non-replicative episomes, which can temporarily express the target gene within a certain period of time [19]. Their immunogenicity, insertion mutation and carcinogenic risk are also relatively lower [17]. Some of these transfection methods have even been approved for clinical use [19]. However, the transfection efficiency is comparatively low, and the protein level encoded by the target gene is correspondingly reduced [15]. Some studies are working on improving the non-viral transfection efficiency and stability by changing the transfection strategy or combining different transfection genes [20]. Both chemical transfection and physical transfection are non-viral transfection strategies. Chemical transfection methods include strategies using cationic polymers, cationic liposomes and inorganic materials as vectors. These vectors combine the plasmids into nanoparticles by electrostatic concentration or encapsulation, due to the effect of electric charges, the nanoparticles are adsorbed on the cell membrane, the cell subsequently incorporates the nanoparticles by endocytosis [21]. However, nucleic acids transferred into cells in this manner have to undergo a second step, that is, the degradation by lysosomes and nucleases, which reduces the transfection efficiency [22]. Transfection methods such as microinjection, electroporation, and nucleofection are classified as physical transfection methods. Electroporation utilizes a transient electric field to induce pores in the cell membrane, and combines the nucleic acid with the cell membrane through charge attraction, and then endocytosis occurs to introduce the nucleic acid into the cell. Microinjection can directly deliver the plasmid into the nucleus by using nanoneedles. This approach can avoid the degradation of nucleic acid by cytoplasmic lysosomes and nucleases encountered in other transfection methods. However, this method is not suitable for transfecting genes into large numbers of cells [21]. Nucleofection is a transfection method based on electroporation, setting specific electric field parameters and suspension formulations to directly deliver plasmid nucleic acid to the nucleus, it combines the advantages of high transfection efficiency and direct nucleic acid delivery to the nucleus. We previously performed nucleofection on fibroblasts and showed that they can still maintain their normal morphology and adhesion ability after 6 days (Fig. 1).

Many researchers have explored the transfection efficiency of nucleofection in fibroblasts and examined the efficacy of the transgenic fibroblasts used for further cell therapy. In this article, we review the published work on fibroblasts nucleofection in recent years, and summarize the different angles of researches, advancements of methods, and evolution of applications.

## Isolation and culture of fibroblasts

Due to different research purposes, different species and tissues have been selected to be used for isolating and culturing fibroblasts for experiments. Some studies have pointed out that the number of passages, confluence state, growth rate, and division phase of fibroblasts before they are used for nucleofection can affect nucleofection efficiency. Although opinions differ on the optimal number of passages for fibroblasts, most studies agree that cells should be kept in a low-passage exponential growth phase prior to transfection. Reaching high confluency should be avoided, since cells in a high confluence state are more resistant to reprogramming. In addition, the composition of the fibroblast culture medium was also indicated to correlate with the final nucleofection efficiency by affecting the proliferation rate of fibroblasts. So far, an overview over experimental design and statistical data on specific effects of these factors on fibroblasts nucleofection efficiency is missing.

### Fibroblast extraction

Fibroblasts have been successfully extracted from diverse species and tissues for research by enzymatic and explant culture methods. The enzymatic method mostly refers to the use of collagenase, dispase, hyaluronidase or trypsin to digest the chopped tissue specimens. The typical explant method consists of cutting the sample into tissue fragments of about 0.5 mm in length, planting them in a petri dish with culture medium, and letting the cells grow out of the sample. For example, we have successfully isolated fibroblasts from the dermis of the skin obtained from the back of Lewis inbred rats by the enzymatic method in previous studies. In these experiments, the first 3 passages of the cultured cells were used for subsequent experiments [11, 13]. Some studies have also successfully extracted fibroblasts from the dermis of human skin by enzymatic and explant culture methods [23–28]. Skrzyszowska et al. and Ko et al. minced the ear skin of 6-month-old and 10-day-old sows, respectively, as tissue explants to culture monolayers of adherent fibroblasts [29, 30]. Skrzyszowska et al. chose to culture fibroblasts for more than 3 passages before using them in subsequent experiments, whereas Jacobsen et al. extracted primary cells and put them into use immediately, or the cells were firstly cryopreserved and then recovered before putting into use. In a study by Zanin et al., sciatic nerves of 8–10 week old rats were taken out and cut into 1 mm length segments for use as explants to isolate fibroblasts [31]. Miki et al. removed islets from brain-dead donors, selected healthy parts and isolated human fibroblasts [32]. Fibroblasts can also be extracted from rat connective tissue, C57BL/6 mouse embryos, zebrafish embryos and Murrah buffalo embryos by enzymatic digestion or explant culture [33–36]. Furthermore, fibroblast cell lines are also often studied as nucleofection targets, such as the human fibroblast cells Hs27 [37], human skin fibroblast cell line CCD-1079Sk [38], human foreskin fibroblast cell line HFF-1 [27], mouse embryonic fibroblast cell line NIH3T3 [39].

### Fibroblast passage and culture

Notwithstanding some studies emphasize the necessity to use cells within three to five passages in the nucleofection process [15], some studies recommend that the number of passages needs to be more than three [29]. However, from the above studies, it is impossible to directly observe the effect of using primary cells with different passages or cell lines with more passages on the subsequent nucleofection process. Perhaps the most suitable fibroblast type or passage number for nucleofection can be explored by the same transfection method for primary fibroblasts or cell lines from different sources and passages. Moreover, Kime et al. pointed out that during the cell culture process before transfection, the cells should be kept at a low passage, maintained in the exponential growth stage, and prevented from reaching high confluence, because when cells proliferate to a highly confluent state, their growth rate decreases while their resistance to reprogramming increases, which may be detrimental to the subsequent nucleofection process [40]. Another research team also expressed a similar view, believing that when fibroblasts grow to 80–90% confluence, they should be passaged at a ratio of 1:5 [40]. Keeping the fibroblasts in the exponential growth stage, where they are actively dividing, allows to make use of the nuclear membrane rupture during the nuclear division, which is conducive to gene reprogramming. Another important aspect is nutrition of the cells. Most studies use Dulbecco's Modified Eagle Medium (DMEM) containing 10–20% fetal calf serum (FCS), 1%-2% non-essential amino acids, 2-4 mM glutamine, 1–1.5% b-mercaptoethanol and 1% penicillin–streptomycin to incubate skin-derived fibroblasts in 37 °C 5% CO_2_ humidified incubators. Some of these studies added basic fibroblast growth factor (bFGF) to the culture medium, but effects on subsequent transfection were not observed. Only Kuebler et al. and Teklemariam et al. chose Iscove's Modified Dubecco's Medium (IMDM) to culture fibroblasts [14, 24], IMDM contains higher concentrations of nutrients and is suitable for high-density cell culture, where fibroblasts can be harvested at higher yields than in DMEM. However, considering the main points of pre-transfection cell culture (maintaining in the exponential growth phase) as mentioned by Kime et al., the fibroblasts in IMDM can proliferate too fast, which may be inappropriate to control their quantity before transfection. The isolated fibroblasts can be stained with phalloidin and DAPI, respectively, to stain the cytoskeleton and nucleus for observation of cell morphology under a fluorescence microscope. In addition, the identification and characterization of fibroblasts can be accomplished by fluorescent staining with antibodies against various markers, such as human fibroblast surface markers Thy-1, tubulin, vimentin, cytokeratin-18 [15, 36, 41] or prolyl 4-hydroxylase beta subunit (P4Hβ) [13].

### Fibroblast nucleofection

#### Pre-nucleofection

There are a few publications reporting that the subsequent nucleofection process is significantly affected by different sources of fibroblasts. Some studies mentioned that primary fibroblasts and NIH3T3 cells are difficult to transfect [42–44], but in many other studies the transfection efficiency of these cells has been satisfactory. It can be achieved by using cells with lower passage numbers and controlling the confluency of the cells (40%-80%) in the culture dishes before transfection as described above to maintain cell viability and mitotic activity [15].

Cell contamination often severely affects transfection outcomes [45]. Bacterial and fungal contamination of cells is usually easily detected during cell culture due to obvious medium turbidity. The main exemption to this rule is contamination with mycoplasma, which may not render the medium turbid, but in most cases, such pathological changes of mycoplasma-contaminated cells are relatively mild, mainly cell proliferation is slowed down. Phase contrast microscopy, electron microscopy, and DNA fluorescence staining can be used to detect the presence of mycoplasma. Once mycoplasma infection is found, mycoplasma-sensitive antibiotics should be added or experimental cells should be replaced.

### Nucleofection

#### Selection of nucleofection protocol and improvement of transfection solution

Nucleofection is an electroporation-based transfection modality with a variety of cell-specific transfection buffers and different programs that control voltage, frequency, and pulse duration. Skrzyszowska et al. used Sacl enzyme to cut the constructed plasmid DNA into linear conformation. They added fibroblast nucleofection buffer for nucleofection of pig fibroblasts, wherein the U-20 procedure was used for pig fetal fibroblasts, U-23 procedure was used for porcine adult dermal fibroblasts. In their work, they only give numbers for different protocols (e.g. U20, U23), while treating the exact procedure as a trade secret. The transferred target sequence carried an enhanced green fluorescent protein (eGFP) gene. This allowed to detect transfection efficiency of the gene by observing the fluorescence intensity of eGFP [29]. Ko et al. carried out nucleofection of porcine fibroblasts using the U-23 procedure. They reported a cell viability of up to 87.9%, and transfection efficiency of 80.8%. This was much higher than cell viability after lipofection (62.0–77.7%), which also had a very low transfection efficiency of 6.1–6.3% [30]. Mehta et al. nucleofected Murrah buffalo fetal fibroblasts using the EN-150 procedure, and the cell viability (53.8 ± 4.2%) and transfection efficiency (73.6 ± 1.4%) were both higher than those using Fugene HD (nucleofection efficiency: 50.4 ± 1.7%, cell viability: 24.6 ± 2.9%) and Lipofectamine 2000 (nucleofection efficiency: 71 ± 1.2%, cell viability: 30.4 ± 3.1%) lipofection [41]. Another study tested the transfection efficiency of 30 different procedures of nucleofection using porcine and rabbit embryonic fibroblasts, of which U-20 was the most efficient for porcine embryonic fibroblasts with a transfection efficiency of 90% and the cells toxicity was only 5%. U-23 was the most effective for rabbit embryonic fibroblasts with a transfection efficiency of 38%. Rabbit embryonic fibroblasts seem to be more difficult to transfect than porcine-derived fibroblasts. This study also tested the transfection efficiency of various chemical media transfection methods, among which the transfection efficiency of Effectene was 18%, Lipofectamine 2000 was 28%, Lipofectamine Plus was 20%, and polyethyleneimine was 32%, which were all significantly lower than the nucleofection efficiency of porcine embryonic fibroblasts, and even lower than the nucleofection efficiency of difficult-to-transfect rabbit-derived cells [46]. Other studies confirmed in human, porcine and mouse fibroblasts that nucleofection is much more efficient than conventional electroporation and lipofection [19, 28, 47, 48].

Previous studies have compared and found that the cuvettes provided by the manufacturer of the Nucleofector device and cuvettes from Eppendorf had no significant difference in the nucleofection process. Under certain circumstances, the transfection solution provided by the manufacturer could even be improved [13]. It was found that using the U-30 procedure for rat fibroblasts and replacing the nucleofection buffer with DMEM supplemented with 10% FCS had the highest transfection efficiency (about 85%) without affecting the growth and proliferation activity of the cells, which was a significant improvement over the transfection efficiency of standard fibroblast nucleofection buffer which only reached 68% in this application. Using the U-24 procedure for human dermal fibroblasts, but replacing the nucleofection buffer with DMEM supplemented with 10% FCS, the transfection efficiency (around 57%) was below that of the standard method of nucleofection (around 79%), but after replacing the standard transfection solution with ITS liquid medium supplement, the transfection efficiency was comparable to the standard solution (about 83%). As Eppendorf cuvettes, DMEM with FCS, and ITS liquid medium supplement are relatively inexpensive, a more economical and efficient nucleofection method was developed [11, 13].

#### Stability of gene integration and DNA damage response

Skrzyszowska et al. compared cell viability and proliferative activity after nucleofection and lipofection, both of which were higher in the nucleofection group. However, in PCR screening results, only in 1 of the 5 analyzed groups of nucleofection, the gene was integrated into the nuclear genome, while the transgenes of 3 of the 5 analyzed groups of lipofection were successfully integrated [29]. Therefore, although the transfection efficiency of nucleofection technology is high, it may have disadvantages in the stability of the integration process of the transgene into the nuclear genome. At the same time, it cannot be ruled out that the target gene construct in this experiment was resulted in the failure of the transgene or the loss of the transgene during the cell cloning process after nucleofection. On the other hand, in the study of Zanin et al., amazing performance of nucleofection in terms of high efficiency and long-term stability was reported using the T-16 procedure to nucleotransfect primary fibroblasts isolated from rat sciatic nerves. The transfection efficiency was much higher than that of lipofection, and the continuous expression duration of the target gene reached 30 weeks, which was also much longer than that of lipofection and even the target gene expression duration in lentiviral transfection in their study [31]. Although nucleofection has the advantages of high efficiency and low cytotoxicity, Huerfano et al. found that the use of U-30 procedure in NIH3T3 cells and mouse embryonic fibroblasts nucleofected with different target genes elicited a strong inducible type I interferon (IFN) response and DNA damage response (DDR). When the same cells were transfected with the same plasmid by means of a cationic polymer (Turbofect), the levels of IFN and DDR were significantly lower than those by nucleofection [39]. The commercial description of nucleofection claims that DNA is "directly delivered" into the nucleus, but Huerfano et al. considered that this "direct delivery" is essentially just the faster transfer of DNA from the cell membrane to the nucleus. The faster transfection rate of nucleofection is also reflected in other studies. Compared with gene expression that starts 24 h after lipofection, nucleofection takes only 3 h [23]. Although not demonstrated, in addition to eliciting a higher degree of IFN, Huerfano et al. observed that nucleofection of different plasmids caused similar inhibition of cell proliferation, which is rarely reported in other studies. Interferons can modulate some cellular physiological and pathological behaviors by participating in many cell signaling cascades. In many studies involving nucleofection, it has not been considered that this process may cause side effects of excessive IFN activation, which may have some influence on the results of these studies. It is speculated that this reaction is caused by the recognition of DNA by cytoplasmic DNA sensors when DNA is delivered in the cytoplasm, during the "rapid transfer" of DNA from the cell membrane to the nucleus. It is recognized by DNA sensors as fracture of DNA and further activates the DDR, another hypothesis is that the elevated ROS levels observed during nucleofection induce IFN responses as well as DDR. Unfortunately, the specific mechanism of DDR and IFN induced by nucleofection has not been verified and elucidated, and further exploration is needed. Kime et al. mentioned in a method guide that the addition of nucleofection solution and cell suspension in strict accordance with the dosage in the instructions, the control of the number of pre-transfected cells, and the control of the transfection operation time are all closely related to the success of nucleofection, the transfection efficiency and cytotoxicity [40].

The implementation conditions and transfection results of nucleofection in related studies are summarized in Table 1.

**Table 1:** The implementation conditions and transfection results of nucleofection in related studies

Cell type	Species	Passage	Cell confluence	Procedure	Cell viability	Transfection efficiency	Reference
Dermal fibroblasts	Rat	2	80%-90%	U-30	> 95.00%	·51.50% ± 7.90% (Standard method) ·80.50% ± 5.00% (Modified method: DMEM + 10% FCS)	[11]
Dermal fibroblasts	Rat	≤ 3	80%-90%	U-30	/	·68.34% ± 10.32% (Standard method) ·85.35% ± 11.56% (Modified method: DMEM + 10% FCS)	[13]
Dermal fibroblasts	Human	≤ 3	80%-90%	U-24	/	·83.88% ± 9.67% (Standard method) ·57.88% ± 3.45% (Modified method: DMEM + 10% FCS) ·79.21% ± 1.62% (Modified method: ITS supplement)	[13]
Dermal fibroblasts	Human	/	80%-90%	U-20	80.00%- 90.00%	40.00%-50.00%	[14]
Dermal fibroblasts	Pig	3–4	/	V-13	/	60.10%	[19]
Dermal fibroblasts	Rat	2–4	/	U-23	91.10%	72.50%	[20]
Dermal fibroblasts	Human	/	/	U-23	37.00%	10.00%	[23]
Dermal fibroblasts	Human	1–5	/	U-30 T-018	> 68.70%	34.60%	[28]
Dermal fibroblasts	Pig	/	90%	U-23 V-13	87.90% ± 7.40% (U23)/ 85.40% ± 7.50% (V13)	·80.80% ± 6.20% (U23) ·86.00% ± 2.50% (V13)	[30]
Sciatic nerve fibroblasts	Rat	/	80%	T-16	/	80.00%	[31]
Dermal fibroblasts	Rat	/	/	U-23 P-22	/	·57.33% (U-23) ·57.00% (P-22)	[33]
Embryonic fibroblasts	Zebrafish	/	/	O-20	30.00%- 35.00%	35.00%-43.00%	[35]
Fetal fibroblast	Buffalo of Murrah breed	/	70%-80%	EN-150	70.00%- 80.00%	73.60% ± 1.36%	[36]
Fetal fibroblast	Buffalo of Murrah breed	2–3	70%-80%	EN-150	53.80% ± 4.20%	73.50% ± 1.40%	[41]
Embryo fibroblasts	Pig	16	/	U-20	95.00%	90.00%	[46]
Embryonic ear fibroblasts	Rabbit	/	/	U-23	/	38.00%	[46]
Embryonic fibroblasts	Pig	3–7	/	U-23	57.80%	79.00% ± 0.80%	[48]
Kidney fibroblasts	Pig	/	/	T-007	/	65.00%-70.00%	[49]

**Fig. 2:**
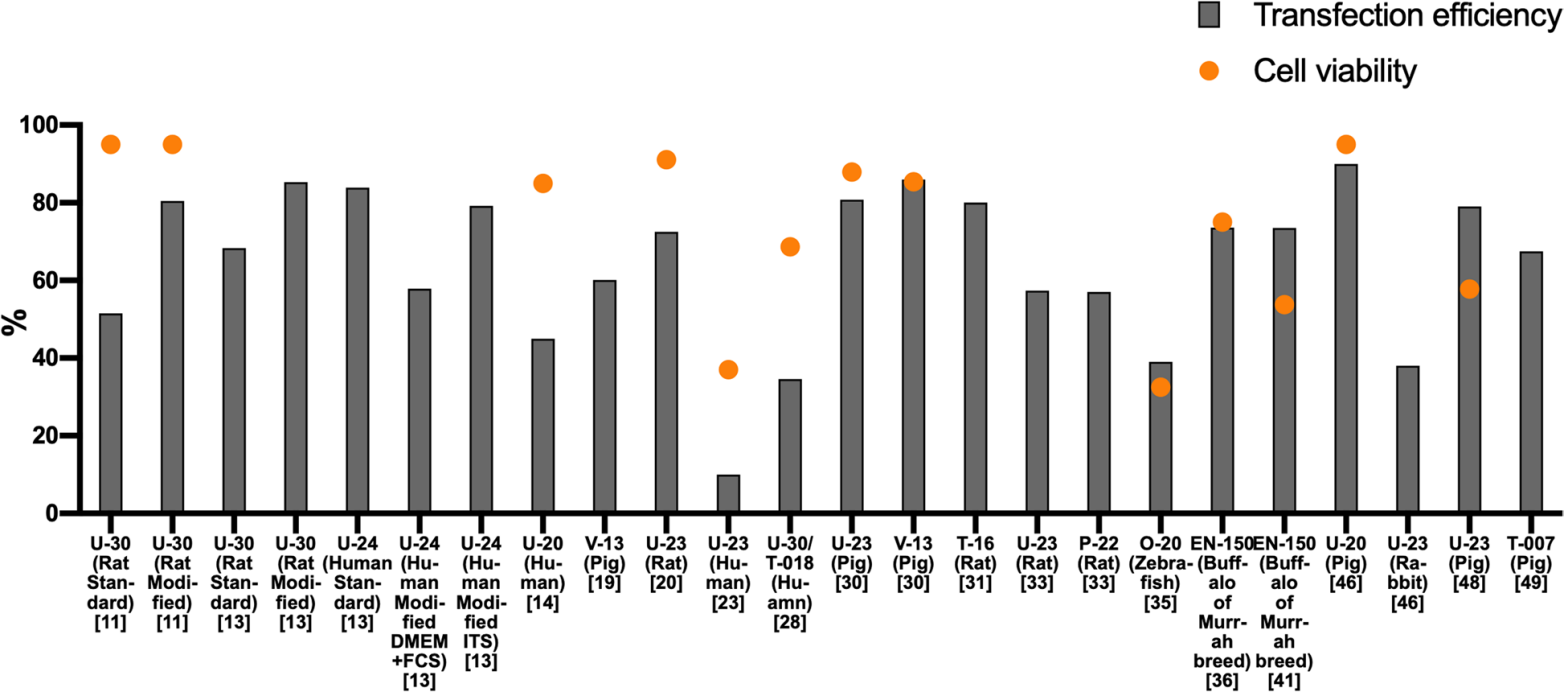
Gray bar represents transfection efficiency, orange dot represents cell viability, the groups with no cell viability value indicate that the cell viability after nucleofection is not mentioned in the original papers. The corresponding references are marked below the program names

In order to more intuitively present the relationship between the sources of fibroblasts, the nucleofection procedures used in each study with the nucleofection efficiency and cell viability in Table 1, we drew a column-scatter chart (Fig. 2).

#### Post-nucleofection

The composition of the nucleofection standard solution is unreported, and may have a certain negative impact on cell survival and proliferation. Most studies stated that the cell viability after nucleofection is higher than other chemical or physical transfection methods, but it is always lower than the viability of untransfected cells. Thus, some researchers recommend that 24 h after nucleofection, the standard solution of nucleofection should be replaced with DMEM, IMDM or other inducible medium according to different experimental purposes [33, 50]. Considering the high efficiency of nucleofection (the target gene starts to translate from 3 h after transfection), Badakov et al. replaced the transfection reagent with culture medium 6 h after nucleofection [35]. Many studies use the pmaxGFP plasmid as a vector for nucleofection experiments. After transfection, flow cytometry or fluorescence microscopy can be used to quantify the transfection efficiency by GFP fluorescence [13, 14, 20, 24, 28, 29, 36, 38, 41, 46, 51]. The transfection efficiency can also be observed and detected with fluorescence microscopy by fluorescent staining against specific marker proteins encoded by the target genes [32, 50, 52]. Using qRT-PCR, Western Blot or ELISA to detect the expression of the transfected target genes can also partially reflect the transfection status, but the expression of the genes is affected by many factors, so it can only be regarded as a partial reference for the success of the transfection [23, 27, 37]. There are some studies that did not use plasmids but siRNA for nuclear transfection of fibroblasts, and then detected the expression of the target genes of the siRNA through qRT-PRC and Western Blot methods [25]. Some researchers aimed to generate human induced pluripotent stem cells through nucleofection from fibroblasts. This process may take 21 days or more and they considered that fibroblasts will become senescent after 14 days of nucleofection, which would affect the experimental results. Therefore, they performed a second nucleofection on days 2–7. Surprisingly, the cells that underwent the second round of nucleofection died in large numbers, even though they were able to obtain pluripotent marker-positive target cells. Some of the selected cell clones were found stopping proliferation and turning to apoptosis within 2 weeks. Perhaps it should be considered whether there is genetic damage to the cells by secondary nucleofection, but this is not yet clarified [27]. The study by Huerfano et al. confirmed that nucleofection does cause IFN and DDR. The content of reactive oxygen species (ROS) in cells undergoing nucleofection increases, and the level of oxidative stress elevates [39], which may cause DNA damage, thereby inhibiting the normal physiological functions of cells. As mentioned earlier, modification of the nucleofection standard solution, or early replacement of the nucleofection solution with culture medium may decrease the stress and reduce this damage to the cells.

## Applications of nucleofection

Gene editing of fibroblasts by nuclear transfection for clinical application is a possible direction of this technology. Although it has not yet been applied to the clinic, related studies on nucleofection of fibroblasts have been carried out in various ways. For example, in research about tissue repair and reconstruction, the genes encoding VEGF165 and bFGF were transferred into fibroblasts by nucleofection, and the cells were injected into the gracilis and adductor muscles of ischemia model rat lower limbs. This significantly improved the circulation reconstruction in this model [11]. Biodegradable scaffolds are often used as reinforcement materials in surgical operations, and can also guide the growth of new tissues. By attaching fibroblasts pre-nucleotransfected with VEGF165 to the scaffold, the gene-modified cells can be successfully targeted into the site requiring tissue repair. This prevents off-target effects, and at the same time can promote the vascularization of the target site and accelerate tissue repair through the high level of VEGF165 protein secretion [37]. Reprogrammed fibroblasts can also be directly infused into the wound site to promote wound healing by expressing pro-vascularization or pro-deposition proteins [19]. Different specific secreted proteins expressed by reprogrammed fibroblasts also have a variety of important applications, such as pre-transplantation culture of islets [32] and promoting the repair and healing of bone defects [20]. The gene encoding brain-derived neurotrophic factor (BDNF) can also be transferred into fibroblasts by nuclear transfection. Such reprogrammed fibroblasts can be delivered to the cochlea with sensorineural hearing loss and support the survival of spiral ganglion neurons there by secreting BDNF, enabling cochlear implants to work successfully [31]. Nucleofection technology also has been studied in embryo engineering. Through nucleofection, specific genes with the ability to enhance the development of porcine embryos were transfected into fibroblasts, and then these transgenic cells were used as nuclear donors for cloning procedures, which successfully resulted in transgenic cloned porcine embryos [29]. Transgenic porcine with knockout of a gene that causes hyperacute xenograft rejection have been generated to successfully transplant porcine organs into primates without causing lethal damage from hyperacute rejection [30]. In addition to the research on the rejection of organ transplantation, there is also a way to deal with the immune rejection caused by allogeneic fibroblast transplantation. This rejection can be avoided by nucleofection to transfer immunosuppressive genes expressed primarily at immune-privileged sites [14]. There are also studies that use nucleofection technology to transfer the human insulin gene into buffalo fetal fibroblasts, and then use it to produce transgenic embryos. This aims at enabling transgenic buffaloes to express human insulin and secrete it in milk, to explore an efficient method for mass production of human insulin [36, 41]. Taking advantage of the potential of fibroblasts to transform into other cell types, genes that induce neuronal differentiation can be transferred into fibroblasts by nuclear transfection. They then can be reprogrammed to promote their differentiation into neurons, which can set the stage for subsequent research on neurodegenerative diseases [50]. Fibroblasts can also be induced to transform into induced pluripotent stem cells (iPSCs) by introducing some specific transcription factor genes by nucleofection, benefiting the research of various human diseases [17, 38, 40, 53, 54]. For example, fibroblasts, isolated from skin biopsies of one female patient with autosomal recessive Alport syndrome (ARAS) homozygous for the COL4A3 mutation and two male patients with X-linked Alport syndrome (XLAS) hemizygous for the COL4A5 mutation, were induced into iPSCs by nucleofection, which provided a very useful resource for studying the pathological mechanism and treatment of Alport syndrome [24, 26]. Turner syndrome is a rare disorder associated with complete or partial deletion of the X chromosome. Fibroblasts were isolated from turner syndrome (45XO) fetal tissue and nucleofected to generate integration-free iPSCs. These TSiPSCs were further modeled for studies on the mechanisms and treatment of turner syndrome [55]. For some recessive gene diseases, the use of nucleofection to introduce genes encoding the normal protein that is missing in the disease can have great therapeutic potential. For example, Friedreich's ataxia (FRDA) is caused by a mutation in the frataxin gene. Increasing the expression of the frataxin gene by nucleofection can lead to beneficial results [51]. In addition, a research team transferred BPV-E4 and BPV-E1^E4 genes into equine adult cutaneous fibroblast cells (ACFCs) by nucleofection, and found that equine ACFCs undergo sarcoid-like tumor transformation, providing new ideas for the clinical treatment of horses with sarcoma-associated neoplasia of the skin and subcutaneous tissue [56]. Fibroblasts play a key role in the occurrence and development of various diseases, so fibroblasts are also an important target for mechanism research, diagnosis and treatment research of corresponding diseases [44, 57, 58]. The delivery of genes into fibroblasts is also one of the basic tools for pathological research of diseases, and plays an increasingly important role in the development of therapeutic methods.

The applications of nucleofection in related studies are summarized in Table 2.

**Table 2:** The applications of nucleofection in related studies

Application purpose	Experi-mental animal	Target cell	Transferred gene	Cell administration	Result	Reference
Treatment of lower extremity ischemia	Rat	Rat dermal fibroblasts	·VEGF165·bFGF	Local injection	Enhanced collateral formation in ischemic areas	[11]
Research of Alport syndrome	In vitro	Human dermal fibroblasts	·OCT3/4·SOX2·KLF4·LIN28·L-Myc·p53 shRNA	Cultured in vitro	Generation of colonies of induced pluripotent stem cells	[24, 26]
Research of systemic sclerosis	In vitro	Human dermal fibroblasts	·c-Jun siRNA·c-Fos siRNA	Cultured in vitro	Identification of c-Jun and c-Fos as TGFβ downstream mediators of the profibrotic effect in systemic sclerosis	[25]
Swine embryo engineering	In vitro	Porcine fetal fibroblasts	·Human growth hormone gene·Rat whey acidic protein promoter	Embryo reconstitution in vitro	Improved embryonic development	[29]
Research of pig-to-primate xenotransplantati-on	In vitro	Porcine fetal fibroblasts	·α 1,3-galactosyltra-nsferase (GalT) (KO)·Membrane cofactor protein (MCP)	Human serum-mediated cytolysis assay	Reduced cytolysis	[30]
Treatment of sensorineural hearing loss	In vitro	Rat sciatic nerve fibroblasts	·Brain-derived neurotrophic factor (BDNF)·Neurotrophic factor 3 (NT3)	Cultured in vitro	·Successful transfection and expressio of BDNF and NT3·Survival maintenance of spiral ganglion neurons	[31]
Improvement of angiogenesis in ischemic flap	Rat	Rat dermal fibroblasts	VEGF164	Local subcutaneous injection in the flap area	Acceleration of neovasculariza-tion	[33]
Production of human insulin using transgenic buffaloes	In vitro	Buffalo fetal fibroblasts	Expression vector for the human insulin gene inserted between the DNA fragments of the promoter and terminator regions of mammary gland-specific buffalo β-lactoglobulin	Embryo reconstitution in vitro	·Successful human insulin gene transfection·Reduced embryonic development	[36, 41]
Research and treatment of neurodegenerative diseases	In vitro	Human foreskin fibroblasts	·Ascl1·miRNA124·p53 shRNA	Cultured with neuronal induction and maturation medium	Successful conversion of human fibroblasts into neurons	[50]
Research of Friedreich's ataxia (FRDA)	In vitro	FRDA patient fibroblasts	pCR3.1-TALE_Frat#8_-TADs	Cultured in vitro	Increased frataxin protein expression and maturation levels	[51]
Research of equine sarcoid	In vitro	Equine adult cutaneous fibroblast cells (ACFCs)	BPV-E4/ BPV-E1^E4	Cultured in vitro	Sarcoid-like neoplastic transformation of equine ACFCs	[56]

## Conclusions

Making use of the characteristics of fibroblasts, nucleofection treatment of fibroblasts has been proven to be of potential in a lot of aspects. In addition to promoting the repair and reconstruction of soft tissue or bone tissue damage, it can also be used to support the survival of adjacent cells through its exocrine function, used as a nuclear donor to produce transgenic animals, induce it into other differentiated cells or iPSCs, etc.. The diverse application modes make fibroblast nucleofection of extraordinary significance in disease research and development of novel treatments. The high efficiency of nucleofection has been well confirmed, whereas the specific and detailed way in which nucleic acids "directly" enter the nucleus is still unknown. To clarify this process in the future may allow to further improve it. Although the details of the nucleofection procedure and standard solution formula are unknown, some methods have been developed to improve the nucleofection process. This can elevate the transfection efficiency while reducing the experimental cost and ensuring cell viability, which undoubtedly paved the way for the development of fibroblast nuclear transfection-related research. However, it should be noted that nucleofection like other gene transfection methods may cause IFN and DDR, affect the level of oxidative stress in cells, and interfere with subsequent experiments. Similarly, it is necessary to study the specific mechanism of cell damage during the gene transfection process, as it can help to find a better way to minimize the negative impact on experimental cells. This can further promote the improvement and development of fibroblast nucleofection to meet the needs of various researches and clinical applications.

## Data Availability

Not applicable.

## Abbreviations

ECM: Extracellular matrix
RDEB: Recessive dystrophic epidermolysis bullosa
DMEM: Dulbecco's modified eagle medium
FCS: Fetal calf serum
bFGF: Basic fibroblast growth factor
IMDM: Iscove's modified Dubecco's medium
P4Hβ: Prolyl 4-hydroxylase beta subunit
eGFP: Enhanced green fluorescent protein
IFN: Interferon; DDR: DNA damage response
ROS: Reactive oxygen species
BDNF: Brain-derived neurotrophic factor
iPSCs: Induced pluripotent stem cells
ARAS: Autosomal recessive alport syndrome
XLAS: X-linked alport syndrome
FRDA: Friedreich's ataxia
GalT: α 1,3-Galactosyltra-nsferase
MCP: Membrane cofactor protein
NT3: Neurotrophic factor 3
ACFCs: Adult cutaneous fibroblast cells
